# Double burden of vulnerability for refugees: conceptualization and policy solutions for financial protection in Iran using systems thinking approach

**DOI:** 10.1186/s12961-023-01041-2

**Published:** 2023-09-11

**Authors:** Saeed Shahabi, Manal Etemadi, Maryam Hedayati, Kamran Bagheri Lankarani, Mihajlo Jakovljevic

**Affiliations:** 1 Health Policy Research Center, Institute of Health, Shiraz University of Medical Sciences, Shiraz, Iran; 2 The National Institute for Health and Care Research Applied Research Collaboration West (NIHR ARC West) at University Hospitals Bristol and Weston NHS Foundation Trust, Bristol, United Kingdom; 3 Population Health Sciences, Bristol Medical School, University of Bristol, Bristol, United Kingdom; 4 Department of Health Services Management, School of Health Management and Information Sciences, Iran University of Medical Sciences, Tehran, Iran; 5 Institute of Advanced Manufacturing Technologies, Peter the Great St. Petersburg Polytechnic University, Saint Petersburg, Russia; 6 Department of Global Health Economics and Policy, Faculty of Medical Sciences, University of Kragujevac, Kragujevac, Serbia; 7 Institute of Comparative Economic Studies, Hosei University, Tokyo, Japan

**Keywords:** Refugees, Healthcare financing, Humanitarian, Systems thinking, Iran

## Abstract

**Introduction:**

Iran is host to one of the world’s largest and longest-standing refugee populations. Although Iran has initiated a basic health insurance scheme for refugees throughout the country since September 2015, the population coverage of this scheme is very low, and various factors have caused a significant percentage of refugees to still lack insurance coverage and often face financial hardships when receiving health services. In response, this study aimed to understand barriers to insurance coverage among refugees in Iran and propose effective policies that can address persistent gaps in financial protection.

**Methods:**

This qualitative study was conducted in two phases. First, a review of policy documents and interviews with participants were conducted to investigate the common barriers and facilitators of effective insurance coverage for refugees in Iran. Then, a systems thinking approach was applied to visualize the common variables and interactions on the path to achieving financial protection for refugees.

**Results:**

Findings showed that various factors, such as (1) household-based premium for refugees, (2) considering a waiting time to be eligible for insurance benefits, (3) determining high premiums for non-vulnerable groups and (4) a deep difference between the health services tariffs of the public and private service delivery sectors in Iran, have caused the coverage of health insurance for non-vulnerable refugees to be challenging. Furthermore, some policy solutions were found to improve the health insurance coverage of refugees in Iran. These included removing household size from premium calculations, lowering current premium rates and getting monthly premiums from non-vulnerable refugees.

**Conclusions:**

A number of factors have caused health insurance coverage to be inaccessible for refugees, especially non-vulnerable refugees in Iran. Therefore, it is necessary to adopt effective policies to improve the health financing for the refugee with the aim of ensuring financial protection, taking into account the different actors and the interactions between them.

## Introduction

According to the United Nations High Commissioner for Refugees (UNHCR), by the end of 2019, 26 million people worldwide had become refugees due to war, violence, human rights violations or other persecutory events [1]. Refugees often present with significant and complex health needs and poorer health outcomes compared with the general population [2]. Several factors during their migration, such as transport in closed containers, accidental injuries, malnutrition and accommodation in detention centres and refugee camps, have a major influence on the health of refugees [3]. Poor access to healthcare services interacts with discrimination and limited social rights, thereby reinforcing exclusion as a root cause of ill health among refugees [4]. This profound change also impacts the sensitive demographic equilibrium between societies in the advanced population ageing stage such as Europe and far younger Arabic, Persian and other Middle Eastern nations [5].

In a universal health system, refugees should be entitled to the same basic benefits as non-migrants, as well as some level of financial protection [6]. Despite equitable aspirations, inequalities between migrants and non-migrants in health and in access to healthcare services persist. Inequalities are the results of the economic situation of migrants, who may lack the means to pay for health services [7]. Given immigrant communities’ vulnerability and barriers to accessing care, it is possible that some of their healthcare needs are not being met [8]. Lack of awareness of one’s rights to healthcare, communication, cultural differences and financial difficulties were reported as the barriers faced by the refugees in accessing healthcare in Asian host countries such as Iran [9]. Furthermore, dynamics of investment in healthcare capacities and infrastructure will be largely influenced by Chinese Belt and Road initiative in decades to come [10].

The WHO emphasizes that on the path to universal health coverage, all people should have access to quality health services without financial hardships. Nonetheless, nearly 2 billion people continue to face catastrophic and impoverishing healthcare expenditures [11]. Financial barriers to healthcare access are significant, owing primarily to a lack of livelihoods, a lack of healthcare insurance, and increased foreigner charges [12]. Notably, financial barriers make it more difficult for vulnerable groups such as immigrants and refugees to benefit from health services than other groups in society. Lack of or insufficient health insurance and delays in obtaining health insurance, which resulted in a medical coverage gap during a critical time, were key barriers that limited refugees’ access to the healthcare system [13].

On the basis of syndemic theory, the synergistic nature of stressors [14], chronic diseases and environmental impacts on immigrant and refugee populations living in vulnerable conditions, we should consider the immigrant experience including migration pathways, poverty, cultural distance and lack of social support [15]. According to the International Organization for Migration, vulnerability is “the diminished capacity of an individual or group to have their rights respected, or to cope with, resist or recover from exploitation, or abuse” [16]. High levels of crowding within refugee households and among those in informal tented settlements, inadequate water supply and sanitation, limited use of masks, inadequate access to healthcare and inadequate community awareness levels are vulnerability factors, and poverty, stigma and fear of legal consequences are contextual factors that further exacerbate this vulnerability [17].

The global evidence of targeting in the health system suggests that those whose disposable income cannot cover basic costs and loan instalment payments are vulnerable [18]. The eligibility criteria for receiving healthcare subsidies determine the government’s perception of vulnerable groups, and consequently, the number of people on subsidies [19]. Given their economic, demographic and geographic characteristics, vulnerable groups are individuals with a lack of adequate access to healthcare services with proper financial support [20].

Iran is host to one of the world’s largest and longest-standing refugee populations. There are nearly a million refugees in Iran, the vast majority of whom are from Afghanistan. Documented migrants and refugees in Iran are covered by the Iran Health Insurance Organization. Indeed, to achieve universal health coverage (UHC), one of the most important indicators of which is universal health insurance, Iran initiated a basic health insurance scheme for refugees throughout the country in September 2015 [21]. According to the published statistics, in the first 2 years of this program in Iran, more than 124 000 refugees were registered in this scheme, and a significant share of them were very vulnerable refugees or refugees with rare diseases [22, 23]. However, the population coverage of this scheme is very low, and various factors have caused a significant percentage of refugees to still lack insurance coverage and often face financial hardships when receiving health services [21]. In addition, a number of challenges are experienced by Iran in providing health insurance for refugees, including the low social and economic status of refugees, the fragile financial capacity of refugees with diseases that are expensive to treat, and the lack of a culture of risk aversion among refugees who do not welcome health insurance [24].

In this Iranian basic health insurance scheme, premiums for the most vulnerable refugees are covered by UNHCR and the government, while other refugees can access the scheme by self-paying premiums [25]. Many refugees are not able to afford the premium costs and can no longer cover their most basic needs if they pay for the whole family premium, which is estimated to represent some 40% of an average refugee family’s monthly expenditure [26]. This lack of insurance and, consequently, high medical expenses result in not referring to medical centres and the unwillingness to be hospitalized [27]. Studies on healthcare access and use by Afghan refugees in Iran demonstrated poor communication with healthcare providers, difficulties with recording refugees’ health data, tradition and culturally related aspects of healthcare-seeking behaviour and some language barriers [28]. Evidence demonstrates high prevalence of both communicable and noncommunicable disorders as well as psychological problems in Afghan refugees and immigrants in Iran. As a consequence of low socio-economic status, poor health condition, improper dietary behaviours and lack of proper immunization coverage, high incidence of physical and mental disorders is expected in Afghan immigrants in Iran, leading to high economic burden and healthcare issues [29].

Reasons for low uptake of health insurance among refugees identified in similar studies around the world include frequent policy changes, lack of a legal instrument enforcing enrolment in the migrant insurance, weaknesses in policy implementation, difficulties in managing the insurance among migrant employers, restriction of insurance benefits to the province and upfront payment of fees for 2 years [30]. Therefore, it is important to know who is recognized as being most vulnerable among a group of vulnerable people in a health system, while also needing to ensure access of the remaining to health services, as it is clear that refugees are vulnerable due to their living condition and the situations they experience before, during and after immigration. All refugees are vulnerable, while some of them are more vulnerable due to their specific disease, which means a double vulnerability as a requirement to eligibility for a premium exemption for refugees in Iran. This definition created some challenges for the so-called non-vulnerable refugees in accessing health insurance, as it is not affordable for them due to their incapacity to pay.

Due to the low number of non-vulnerable people in Iran who have health insurance coverage and the important role insurance coverage plays in access to health services in the Iranian health system, the goal of this study was to find out what stopped non-vulnerable refugees in Iran from getting insurance, assess the vulnerability indicators and come up with policies that can help close gaps in financial protection.

## Methods

The qualitative study was designed and carried out in two phases. First, reviewing policy documents and interviewing participants were conducted on the basis of the Critical Appraisal Skills Programme (CASP) Qualitative Checklist in the first half of 2022 in Iran [31]. Second, a systems thinking approach was applied to visualize the common variables and interactions in the path of achieving financial protection for refugees in Iran. The Institutional Review Board (IRB) of Shiraz University of Medical Sciences (SUMS) was reviewed and confirmed before initiating the study (IR.SUMS.REC.1401.428).

### Phase I

#### Sampling approach

In the first stage, to find policy documents related to the financing and insurance of health services for refugees in Iran, the websites of the Ministry of Health and Medical Education (MoHME), the Ministry of Cooperatives, Labor, and Social Welfare, the Iran Health Insurance Organization, the Social Security organization, and the Islamic Studies Center of the Majlis (Parliament) were manually searched using a number of keywords such as vulnerability indicators, refugees, immigrants, health financing and insurance coverage. In the second stage, recruiting the potential participants was done using both purposive and snowball sampling approaches. In this regard, the research team prepared a list of stakeholders in the field of financing and providing health services to refugees in Iran. Then, the invitation, which contained general information about the research and the research team as well as the research objectives, was sent to potential participants via email or WhatsApp. The individuals were asked to read the form carefully and, if they wanted to participate, give their consent to the interview. In this informed consent form, the individuals were also guaranteed that their identity would remain unknown at all stages of the study and that they were free to withdraw from the study at any stage. At the end of each interview, the interviewee was asked to introduce someone who could provide more information related to the purpose of the study.

#### Data collection

In respect to reviewing the relevant policy documents, only policies that addressed vulnerability indicators in some way when financing and providing health services for refugees were considered. Therefore, the desired information was included in the pre-prepared table, including the name of the law and indicators of vulnerability in Iran’s health system. In the second stage, individual semi-structured interviews were conducted by the first author (a woman with a scientific and executive background in the field of health services financing in Iran) in a quiet environment at the participants’ workplaces. In addition to the written informed consent, the interviewee was told verbally what the goals of the study were before the interview began. Once the interviewee agreed, the interview began. To facilitate the guidance of the interview flow, an interview guide containing open questions was used, including the following questions: (1) What are the vulnerability indicators for the insurance coverage of refugees in Iran? (2) How do these indicators affect the insurance coverage and benefits of health services for refugees in Iran? and (3) What are your proposed solutions to improve financial protection and assist refugees in accessing healthcare? In addition to the audio recording of the interview sessions, the interviewer also used note-taking to facilitate the data analysis process. At the end of each session, the recorded audio file was written and saved with a pseudonym in Word Office software.

#### Data analysis

Thematic content analysis was used to analyse the raw data simultaneously with the data collection [32]. In this way, three authors (M.E., S.SH., and M.H.) repeatedly read the written texts, and after identifying the meaning units, assigned codes to them. Then the merged codes were categorized and placed in two main themes, including challenges and policy solutions. Any disagreement among the authors at this stage was resolved through discussion and, if necessary, with the participation of the expert author (K.B.L.). The analysis process was done manually, and several authors with different research backgrounds were involved to maximize the strength of the findings.

#### Trustworthiness of findings

Credibility, confirmability, transferability, authenticity and dependability are considered the main dimensions of rigour and trustworthiness of qualitative findings [33]. Therefore, a number of strategies were employed to promote the rigour and trustworthiness of the findings, including: evaluating the findings by relevant experts (credibility); reviewing and checking the findings by participants (confirmability); considering the highest diversity through the sampling process (transferability); inserting direct quotes from most of the participants (authenticity); and involving several authors with different backgrounds in data analysis (dependability).

### Phase II

#### Systems thinking approach

Scientific evidence has always taken into account how complex and changing health systems [34] are when trying to find effective ways to improve the health of societies in a fair and efficient way [35, 36]. In this regard, the Alliance for Health Policy and Systems Research, based on the WHO, released a flagship report about applying systems thinking to strengthening health systems, especially in low- and middle-income countries [35, 37]. This report and other similar studies have always emphasized the necessity of using a systems thinking approach in formulating programs to solve social and health problems [38]. Systems thinking is indeed a holistic method for recognizing how the different components of a system interact with each other and is an acceptable approach to proposing an effective problem-solving strategy by identifying powerful leverage points [35, 38, 39].

By using a systems thinking approach, the relationships and interactions among various components are represented by causal loop diagrams (CLDs) to create system maps [40]. In the CLDs, positive arrows (blue arrows) demonstrate a direct causal relationship, while negative arrows (red arrows) indicate an adverse causal relationship between the two interested variables [39, 41]. Specifically, CLDs include reinforcing loops (feedback loops that advance change), which are revealed with an “R,” and balancing loops (feedback loops that resist change), which are revealed with a “B” [40, 42]. Whether the loop is clockwise or anticlockwise is determined on the basis of the general direction of the loop. In this study, the CLDs were made with Vensim PLE 8.1 software (Ventana Systems, Harvard, USA) on the basis of the results of the qualitative stage.

## Results

### A review of vulnerability indicators in upstream documents

The upstream laws in the fields of social welfare and health have determined the indicators of vulnerability. Table 1 summarizes the main laws and their indicated criteria for vulnerability. The most stressed groups of the laws have been related to economic situation, including low-income deciles and the poor; the status of illness and physical injury, including disability and agelessness; and specific groups, including the elderly and children.

**Table 1:** Vulnerability indicators in upstream documents in Iran

Name of law	Main factors of vulnerability
Regulations on how to identify the poor. Regulations, Note 1, Article 14 of the Law on Public Health Insurance	(A) Poor disabled people who do not have the financial ability to live their life and their dependents
	(B) Poor women-headed families and orphan children, the subject of the law on providing for orphaned women and children, approved in 1992
	(C) Families of poor prisoners during the imprisonment of the head of the family
	(D) Victims of natural and unnatural events who have lost their basic means of living or means of employment until they get their basic means of employment or means of employment
	(E) Subjects of the Shahid Rajaei plan (the poor rural elderlies)
	(F) Other cases that are not included in the above clauses and are covered by assistance organizations such as the Imam Khomeini Relief Committee (IKRC) or the country’s welfare organization or should be covered by them, as determined by IKRC
Constitution	Principle twenty-one
	2 – Supporting mothers, especially during pregnancy and child custody, and supporting orphaned children
	4 – Creation of special insurance for widows and elderly and orphaned women
Regulation of social welfare safety umbrella	The priority of providing services to families with absolute poverty will be as follows
	(A) Unsupervised children
	(B) Women-headed households
	(C) Elderly people
	(D) Disabled people
	(E) Others (unemployed, addicts, chronic physical and mental patients, etc.)
General policies of social security	Support for social service target groups, including the homeless, the disabled and the elderly
General policies of health	Residents of underprivileged areas, poor strata and low-income deciles

The Executive Regulation of Health Insurance Coverage for Foreigners Living in the Country, subject to the approval of the Board of Ministers, has introduced the vulnerable beneficiaries as foreigners who are suffering from severe or incurable diseases or households whose economic and social conditions make them vulnerable. The vulnerability includes dual economic and health criteria. The Ministry of Interior has announced the indicators through an instruction to its provincial offices, on the basis of which they declare the eligible refugees to the health insurance offices.

### Findings of qualitative interviews

In total, 17 experts have participated in the study. Their attributes have been described in the Table 2.

**Table 2:** Summary of interview participant characteristics

Organization affiliated to	Interview participant number (%)
Iran Health Insurance Organization (IHIO)	8 (47%)
MOHME – Deputy of Hygiene	1 (6%)
MOHME – Deputy of Treatment	2 (12%)
Ministry of Interior	1 (6%)
UNHCR	2 (12%)
Red Crescent Society	1 (6%)
Academia (medical universities)	2 (12%)

## Vulnerability indicators for refugees

The desired indicators for the inclusion of refugees in the vulnerable group are a combination of health indicators and economic indicators. Health indicators include impoverished and costly diseases such as special and incurable diseases, and economic indicators, due to the lack of statistics and information related to the income level of refugees, are focused on the aggravating or underlying factors of poverty such as homelessness and disability.

To determine whether a refugee is vulnerable, both health status and socio-economic status are considered. This means that a person whose caretaker is workless, has a special disease, has many children, etc., is considered vulnerable. People who also have incurable diseases are considered vulnerable because even if they are rich, they will become poor [Participant 01].

The authorities for determining their vulnerability are the Ministry of Interior and the General Directorate of Refugees of the provinces, and generally, there are people who are sick, have incurable diseases within their families, are vulnerable, and we cover them [Participant 05].

The method of identifying vulnerable refugees is focused on self-targeting. In this way, people enter the website designed by the Ministry of Interior and mark the default indicators of vulnerability and upload related documents. In this way, new people with vulnerability indicators will have the opportunity to express themselves as vulnerable people every year.

There is a system where refugees can upload documents related to their vulnerability. Every year, these documents are reviewed, and those who qualify are recognized as vulnerable refugees. Of course, vulnerability is not only related to people’s health status. Women with addicts or disabled husbands, women-headed households, people over 60, and patients are among those who fall into this category. We also consider people with special diseases such as haemophilia, thalassemia, cancer, MS, etc. as vulnerable [Participant 04].

Thus, any household that does not fall within the scope of the government’s vulnerability indicators will be considered non-vulnerable, and it is assumed that the household has access to a job and income and is able to pay insurance premiums.

According to the United Nations High Commissioner for Refugees and the Ministry of Interior of Iran, a “non-vulnerable” refugee means that they have sufficient resources for their own treatment and sustenance. In other words, the family head is healthy and employed, or they have other healthy family members who have income and live like other citizens of Iran, and typically, they don’t live in the camps. Refugees who live in the camps are generally vulnerable [Participant 01].

Since asylum inherently brings with it a level of vulnerability, it is not possible to assume the ability to pay the insurance premiums of all family members at once at the time of enrolment. Since vulnerable refugees are exempted from paying insurance premiums due to the limited budget of the United Nations High Commissioner for Refugees (UNHCR) and the Iranian government, a level of prioritization takes place to enjoy this exemption on the basis of the inclusion indicators. That is why the beneficiaries enjoy these benefits up to a certain limit. Therefore, there will always be refugees who are vulnerable, but due to the full ceiling of the vulnerable refugees’ number, they have to pay the entire cost of the insurance premium out of their own pockets; otherwise, they remain out of insurance coverage. In this situation, with the financial introduction of the Ministry of Interior or the UNHCR and on a case-by-case basis, the necessary assistance is granted to individuals.

When we reach the cap for covering vulnerable refugees, we can no longer cover anyone. We have at least three times the number of people who are really vulnerable, and the rest are also vulnerable up to 85% [Participant 01].

Many of the refugees who are considered non-vulnerable in terms of terminology are actually vulnerable. This categorization has been defined because only a limited number of refugees can be targeted for premium exemption. It’s because UNHCR and the domestic government’s budget line are limited, and we can only cover this many people with an exemption [Participant 14].

### The challenges of paying insurance premiums for non-vulnerable refugees

1. Considering household size in calculating the premium

Since refugees usually have large households, and insurance coverage depends on meeting the condition of household size, meaning covering all family members simultaneously, it is difficult for them to pay the insurance premiums of households with a large number of family members, who are usually employed in informal and low-income jobs or are even unemployed.

Some refugee households have seven to eight members. In fact, many refugee households are crowded, and since insurance premiums are calculated based on the number of household members, most non-vulnerable refugees do not welcome such insurance plans [Participant 02].

Although some participants believed that it is due to Iran’s special economic situation caused by economic sanctions and the drop in the value of the currency, refugees are actually able to pay their insurance premiums to some extent due to the currency difference and the higher monetary base value of their country of origin. Ultimately, unwillingness to be insured is the main reason for the low rate of insurance coverage among non-vulnerable refugees.

We think that these refugees are not in a very severe financial situation, and considering the value of the currencies of Iran’s neighbouring countries, I think many of them are able to pay their insurance premiums. We see this in the services they receive; we see that they can really afford insurance. Unless households are extremely populous and their financial situation is dire, the majority of people can afford [Participant 06].

2) Assigning a waiting period for non-vulnerable refugees to use their insurance coverage

Insurance companies use a waiting period before people can use their health insurance benefits. This is done to prevent “adverse selection,” which is when people sign up for insurance coverage when they need it the most. This can happen with optional insurances, such as refugee health insurance. Since this type of insurance requires a one-time payment of the entire insurance premium for the entire household at the time of enrolment, many households prefer to take up insurance coverage when they need to receive service, which is one of the types of moral hazard that causes financial pressure on the insurance organization.

To benefit from health insurance services, we consider a 10-day period for non-vulnerable refugees. This means that they cannot use insurance services for 10 days from the day they pay the insurance premium. This policy is to encourage this group, which has a high risk, to be insured in advance. Typically, refugees don’t get insurance when they are not sick, and those 10% of our paid refugees are all sick [Participant 02].

The insurance system is based on principles, the most important of which is the principle of resource pooling and risk distribution. This means that based on the mechanism of compulsory insurance, people are enrolled in the insurance system through prepayment, resources are distributed among different social groups through cross subsidies and people use insurance benefits when they get sick. Enrolment when you need health services is against the principles of insurance.

If only a small percentage of non-vulnerable refugees are to be voluntarily declared and insured, this is a completely non-insurance approach. Out of about 800 000 refugees, only 10 000 people insure themselves every year. This same small group has taken out insurance coverage because their medical expenditures are high [Participant 05].

On the contrary, due to the costs of paying insurance premiums, the rate of abuse and fraud by using other people’s insurance coverage is high, and one insurance coverage is used for several people in the same family and community.

I have seen several cases where people use the same insurance number due to the similarity in appearance and face, particularly in camps. Even now, when the services are provided electronically with a national code for girls, several girls receive service without anyone noticing. Same about boys, elderlies, and so on! [Participant 12].

3) Determining high insurance premiums for non-vulnerable refugees

The rate of insurance coverage among non-vulnerable refugees is very low, and meanwhile, those who register are all sick and expensive cases. Therefore, to comply with the principle of balance of resources and costs, the insurance system is forced to determine the per capita cost of the service package and is not able to reduce the premium rate.

When a small percentage of non-vulnerable refugees purchase insurance, the insurance system is forced to charge higher premiums for each insured to cover its costs. For example, if instead of 100 000 people, 800 000 people are insured, the insurance system can consider a lower premium [Participant 03].

4) Deep tariff gap between the public and private health sectors

Due to the deep tariff gap between the public and private health sectors in Iran and the limitation of basic insurance coverage to the governmental tariff, it is difficult for refugees and Iranians to pay the tariff difference in the private sector in the absence of supplementary insurance. Furthermore, some services are not covered by basic insurance, and their cost must be paid 100% from the patient’s pocket, which will be difficult for refugees.

Many refugees cannot afford to receive health services from the private sector because the money they have to pay is more than what the insurance organization pays. Not only for refugees but for all citizens of Iran, the tariff difference between public and private sector services is very high. When receiving public sector services, 20 to 30 percent of the costs must be paid by the service recipients themselves, especially in relation to medicines, where the amount of co-payment is much higher! The same is true for out-of-coverage services in public places [Participant 05].

## Policy solution to financial protection of non-vulnerable refugees in Iran

1. Expanding vulnerable refugees’ insurance coverage

Seeking support to increase the financial resources allocated to refugees living in Iran through the UNHCR to increase the number of people exempt from paying insurance premiums is one of the proposed policies for the financial protection of refugees. However, no matter how much the beneficiaries increase, there will still be some who, despite their inability to pay, are non-vulnerable and must pay the insurance premium out of pocket.

One of the potential solutions is to increase the number of vulnerable refugees. In recent years, such an approach has started. But more efforts are needed to cover a higher percentage of vulnerable refugees [Participant 01].

- (2) Removing the condition of household size from the calculation of the insurance premium

It is thought that it would help this group if insurance coverage for non-vulnerable refugees, especially those with big families, did not depend on the size of their household.

The insurance organizations should change their policies so that this condition of household size is not considered. This policy makes many refugees reluctant to be insured [Participant 02].

If they remove the condition of the size of the household, they will certainly welcome it. Well, their economic conditions are worse than ours. It is true that many of us do not have good conditions in the current economy of Iran, but in any case, their situation is worse than ours… [Participant 04].

- (3) Reducing the insurance premium rate

The per capita insurance premium of non-vulnerable nationals is twice that of Iranian nationals, and its reduction helps to expand insurance coverage by making it affordable for them, especially in the household dimension.

We raised the amount so that we can’t get anything. I think the best thing is to lower the insurance premiums like the Iranians. It is suggested that they be encouraged to insure themselves [Participant 11].

- (4) Receiving monthly insurance premiums from non-vulnerable refugees

The interviewees believed that different policies, such as changing the time basis of insurance premiums and approximating it to the current situation of insurance coverage for official jobs such as government employees on a monthly basis, can help expand and develop insurance to increase the ability of these groups to pay.

Take, for example, monthly insurance premiums; there is also an electronic system that renews them monthly... I believe that you should not work in such a way that it is an adverse selection that only the sick person comes to insure herself, but I think that a set of mechanisms should be considered so that these people come to insure themselves. What does the social security insurance program do now? It deducts the premium from the salary. It is true that designing for refugees is somewhat more difficult because there is a risk of payment evasion. However, it’s manageable [Participant 10].

## Mapping interactions that surround the financial protection of refugees

**Fig. 1:**
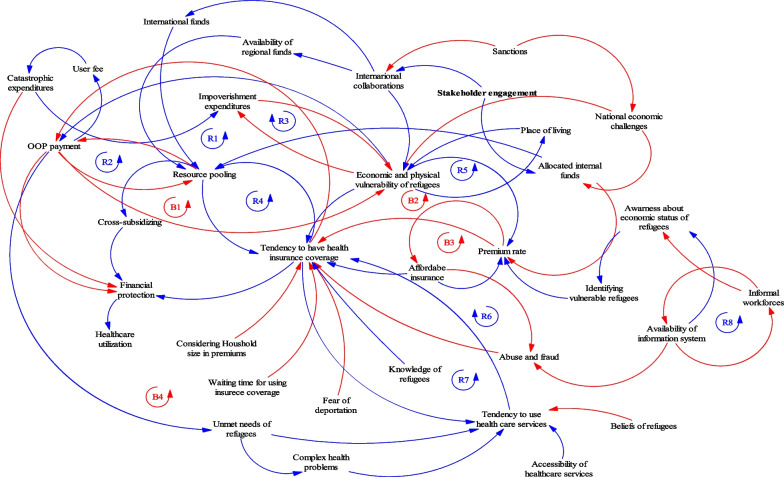
Casual loop diagrams representing various variables and interactions in financial protection of refugees in Iran

Figure 1 indicates the potential variables and pathways that affect the financial protection of refugees in Iran. In fact, the CLDs address several factors and feedback loops that may either strengthen or weaken financial protection when refugees receive health services. As seen in Fig. 1, there are a number of reinforcing loops (R1–R8) and balancing loops (B1–B4). However, it should be noted that the number of loops is higher, but to not make it difficult to check the map, only these loops, which are more important, have been specified in the figure.

R1 reveals how the desire to have insurance coverage can lead to lower out-of-pocket (OOP) payments. This issue causes resources to pool better by entering the insurance mechanism. Further, as shown by R2, the more effective resource pooling is, the more likely it is to reduce OOP payments. On the contrary, paying OOP is associated with an increase in user fees, catastrophic costs, and then impoverishment costs, which can ultimately lead to the deterioration of the refugee’s economic situation and their capacity to pay OOP (B1). B3 shows that there is an inverse relationship between premium rates and affordable insurance. Therefore, it is necessary to consider the factors affecting the insurance premium rate, which have been mentioned in the map more carefully. Notably, the figure highlights the significance of international collaborations in establishing regional collaborations and providing financial resources for Iranian refugees. Another important variable that affects the tendency of refugees to have insurance coverage is their willingness to receive health services (R7). As shown, several proximate factors influence the willingness of refugees to use health services, including unmet needs of refugees, complex health problems, accessibility of healthcare services, and refugees’ beliefs.

## Discussion

The present study reported that various factors, such as (1) considering the size of the household in the calculation of insurance premiums, (2) determining a waiting period to benefit from insurance coverage, (3) determining high premiums for non-vulnerable groups and (4) a deep difference between the tariffs of the public and private sectors in Iran, have caused the coverage of health insurance for refugees, especially non-vulnerable ones, to be unacceptable. Furthermore, some policy solutions were identified to enhance the health insurance coverage of refugees in Iran, such as extending the coverage of vulnerable refugees, removing the household size through calculating premiums, reducing the current premium rates, and receiving monthly premiums from non-vulnerable refugees. Nevertheless, the systems thinking map demonstrated that to achieve financial protection, one of the main goals of UHC, it is essential to consider a wide range of variables and interactions. So, focusing on the identified leverage points shown on the map could help a lot with making and carrying out effective policies to protect refugees’ finances when they use health services in Iran. Comparable challenges of a scale in the efforts to establish UHC were visible across South Asian health systems [43, 44] and in particular leading emerging BRICS economies [45]. There is convenient room for opportunity for Iran to learn from other nation’s mistakes and bottleneck inefficiencies given its intention to join BRICS+ economic association as we approach 2030 [46].

Our findings showed that various factors cause refugees to not have adequate financial protection when receiving health services in Iran. In fact, despite Iran’s significant efforts to establish a health insurance scheme for refugees, a sizeable proportion of refugees remain uninsured after more than 7 years [21]. Considering vulnerability indicators in determining the insurance premium rate for refugees has been one of the most important obstacles to insurance coverage, especially for non-vulnerable refugees. In this regard, refugee groups that are known as vulnerable groups on the basis of predetermined economic and physical indicators do not pay any premiums, and their insurance premiums are paid by the Iranian government or international organizations such as the UNHCR. As a result, a significant share of the people covered by this insurance scheme are vulnerable refugees. Furthermore, many non-vulnerable refugees do not want to participate in such an insurance scheme due to the high premium rate. Indeed, the premium rate for refugee health insurance is determined on the basis of the number of family members [22], and since many Afghan refugees living in Iran have large families, this health insurance cannot be affordable for them. In addition, insurance coverage in this program is considered for at least a 12-month period for registered refugees, and since many refugees come to Iran seasonally and are also unregistered, they are not able to receive such insurance coverage [21, 22, 47]. Such conditions cause the refugees to pay the costs of health services mainly through OOP payments [48], which will eventually lead to their unwillingness to receive services or face catastrophic health expenditures. In line with this finding, Akik et al. discovered that changes in policies regarding Syrian refugees’ vulnerability status in Jordan and Lebanon have increased OOP payments for Syrians [49]. Further, Alawa et al. reported that the lack of adequate funding made nearly 80% of Syrian and Lebanese patients with cancer unwilling to receive health services [50]. Therefore, the vulnerability indicators which the Iranian government, with the cooperation of UNHCR, has used to exempt the refugees from health insurance premiums, despite the diversity of criteria, need to be revised to include more specific needs and groups of refugees, including youth, children and women [16, 51, 52] who are already known to be vulnerable groups of refugees who needs special attention.

In the present study, it is suggested that the level of insurance coverage for refugees should be increased by means of insurance premium rates set for non-vulnerable refugees being reduced. However, it should be noted that the implementation of such policies requires more financial resources from the host governments and international institutions [23, 53]. Interestingly, although financial resources from international organizations have increased (from about 18 billion US dollars in 2013 to more than 27 billion US dollars in 2017) worldwide, the demand for health services from refugees following wars and conflicts has surged [54]. Overall, host governments, international institutions and OOP payments are considered to be the three main sources of funding for refugee health services globally [23]. Nonetheless, considering limited international resources as well as refugees’ ability to pay, the financial burden of providing health services to refugees is mainly imposed on the host countries. Such a situation can be seen in Jordan [55], where the financial burden of providing health services to refugees was so high that the Jordanian government had to change its policies and stop providing free health services to Syrian refugees in 2014 [56]. Iran, as one of the largest hosts of refugees in the world, faces a significant limitation of financial resources in its health sector due to international sanctions and economic problems [57–60]. In addition, wars and internal conflicts in Iran’s neighbouring countries have caused the refugee population to increase significantly, especially after the fall of the Afghan central government. All these factors together have caused the financial burden of providing health services to refugees to double on Iran’s health system. Notably, the sanctions imposed against Iran have caused banking transactions between countries to be severely disrupted [59, 60]. As a result, even refugees with financial resources are not able to benefit from their financial capacity to pay for health expenses. To solve this, effective strategies should be explored to make remittances flow more easily and fluidly [23].

Although the government of Iran has tried since 2015 by creating the refugee health insurance program to provide the necessary opportunity for the integration of refugee health financing in the national financing system and the combination of national and international funds [21], it is necessary to use innovative approaches to strengthen the health financing of refugees. In this regard, creating a regional insurance mechanism for health financing for refugees would significantly escalate financial resources and facilitate service delivery. Such a mechanism can be created by the Organization of Islamic Cooperation (OIC) under the title “Islamic Fund for Refugee Health Financing” and financed by receiving contributions both from member countries and cash donations. The OIC, which is formed with the participation of 57 countries from four continents, can provide significant financial resources to provide health services to refugees in member countries, including Iran [61, 62]. There are several religious Muslim finance tools, such as *zakat* (giving a portion of one’s wealth to charity), *waqf* (assets held for charitable purposes) and *sukuk* (bonds), which can be considered as one of the fund’s financial sources [63]. Similar funds have been established in other regions of the world to help countries in crisis, such as the African Risk Capacity (created by the African Union) [64] and the Caribbean Catastrophe Risk Insurance Facility (supported by the World Bank and other donors) [65]. The creation and development of such funds can facilitate resource pooling and provide the necessary capacity for pre-emergency financing, a paradigm shift that is always emphasized by studies in this field [23]. It is possible to transfer the risk of refugees from host countries to other institutions using such a strategy, so that the burden of financing refugee health services does not fall solely on host countries [55].

### Limitations

Although this study succeeded in exploring the perspectives of various stakeholders related to the financing of health services for refugees in Iran, the perspectives of the refugees themselves were not explored. Therefore, it is necessary for future studies to examine the views of refugees regarding the challenges of financing health services in Iran.

## Conclusions

Several factors, such as considering household size in the calculation of insurance premiums and assigning a waiting period for using insurance benefits, have made insurance coverage of refugees, especially non-vulnerable refugees, unsuitable in Iran. As a result, a significant percentage of them face financial hardships when receiving health services. In response, it is necessary to adapt some effective policies to strengthen the insurance coverage of refugees in Iran, including extending the coverage of vulnerable refugees through international funds, removing household size from premium calculations, reducing the current premium rates and receiving monthly premiums from non-vulnerable refugees. Moreover, different actors and the interactions between them should be taken into account.

## Data Availability

The datasets used and/or analysed during the current study are available from the corresponding author on reasonable request.

## Abbreviations

UNHCR: United Nations High Commissioner for Refugees
UHC: Universal health coverage
CASP: Critical Appraisal Skills Programme
SUMS: Shiraz University of Medical Sciences
MoHME: Ministry of Health and Medical Education
CLDs: Causal loop diagrams
OOP: Out-of-pocket
